# Study on Interference Connection Based on Shape Recovery of NiTiNb Shape Memory Alloy

**DOI:** 10.3390/ma14092328

**Published:** 2021-04-30

**Authors:** Haojie Niu, Yubin Sun, Chengxin Lin, Yutang Zou

**Affiliations:** 1 Department of Mechanics, Dalian Maritime University, Dalian 116026, China; nhj@dlmu.edu.cn (H.N.); sunyubin@dlmu.edu.cn (Y.S.); 2 Department of Marine Engineering, Dalian Maritime University, Dalian 116026, China

**Keywords:** interference connection, NiTiNb shape memory alloy, interference fit size, interference force

## Abstract

Interference connection is an effective method for improving the fatigue life of bolt connections. In this paper, a new method of interference connection was designed based on the shape memory effect of shape memory alloy. Using the method of numerical simulation, a finite element model was established to analyze the stress–strain rule of the bolt and the hole wall under different interference fit sizes. The results show that the stress concentration is formed at the orifice of the connecting plate. When the interference fit size is less than 1%, the connection hole has elastic deformation. When the interference fit size is 1.5%, the hole wall has plastic deformation. When the interference fit size is 2.5%, the maximum stress on the connecting plate is close to the tensile limit of the material. If the interference fit size continues to increase, the strength of the connection structure will be damaged. The connection experiments with different interference fit size were designed, and the interference force was calculated by the pull-out force. The experimental results were compared with the numerical simulation results. The change trend of the interference force with the interference fit size is consistent, which verifies the rationality of the finite element simulation.

## 1. Introduction

As an important mechanical connection, bolt connection is widely used in connecting the important parts of aircraft, such as the fuselage, wing beam, and joint [1]. It is easy for stress concentration to form near the bolt hole of a bolt connection, which can reduce the fatigue life of the connection structure. In order to enhance the fatigue resistance of structural parts and improve their fatigue life, it is necessary to strengthen the weak parts [2]. As an effective fatigue strengthening technology, interference connection has been widely used in the aircraft manufacturing industry. The interference connection can strengthen the key parts of the structure without increasing the weight of the connection structure or changing its form and material. Compared with the ordinary connection, due to the interference prestress, it can significantly reduce the stress concentration at the edge of the hole, strengthen the fitting surface, inhibit the initiation of fatigue cracks, and thus improve the fatigue life of the connection structure, which has good economy [3].

At present, the installation methods of interference connection mostly adopt forced installation, such as hydraulic pressure and the rivet hammer. For structural parts with a larger interference fit size, it is necessary to adopt freezing technology for fasteners and develop specific tooling, and the installation process is complex. These methods have the following shortcomings: low interference fit size, non-uniform interference, and damage to structural parts during installation.

In view of the defects of a traditional interference connection, a new method of interference connection using shape memory alloy (SMA) was designed in this paper. Shape memory alloys (SMAs) are new functional materials that were developed in the early 1960s [4]. Due to their unique shape memory effect and superelasticity, they are widely used in biomedical [5], aerospace [6,7], and mechanical manufacturing [8,9]. NiTi-based SMAs are the earliest developed and most comprehensive SMAs, with the advantages of good shape memory properties, good metal fatigue resistance, high strength, and excellent biocompatibility [10]. They are the most widely used SMAs. The shape recovery temperature of NiTi SMAs is much lower than that of normal atmospheric temperature due to its low transformation temperature. In order to avoid automatic recovery at normal atmospheric temperature, parts made of NiTi SMAs often need to be stored in a liquid nitrogen environment. A new Ni_47_Ti_44_Nb_9_ SMA (if not otherwise specified, all NiTiNb in this paper is Ni_47_Ti_44_Nb_9_) with wide hysteresis was formed by adding the third element, NB, into a NiTi matrix. NiTiNb SMA has a characteristic deformation temperature and the best transformation hysteresis, and strain recovery rate can be obtained by a pre-deformation of 16% at $M_{s}+30$ °C temperature. NiTiNb SMA was pre-deformed at a characteristic temperature, the maximum phase transformation hysteresis could reach 150 °C, and the recovery temperature is much higher than normal atmospheric temperature [11]. The parts made of NiTiNb SMA can be stored and transported at normal atmospheric temperature, which effectively solves the defects of NiTi SMAs.

**Figure 1:**
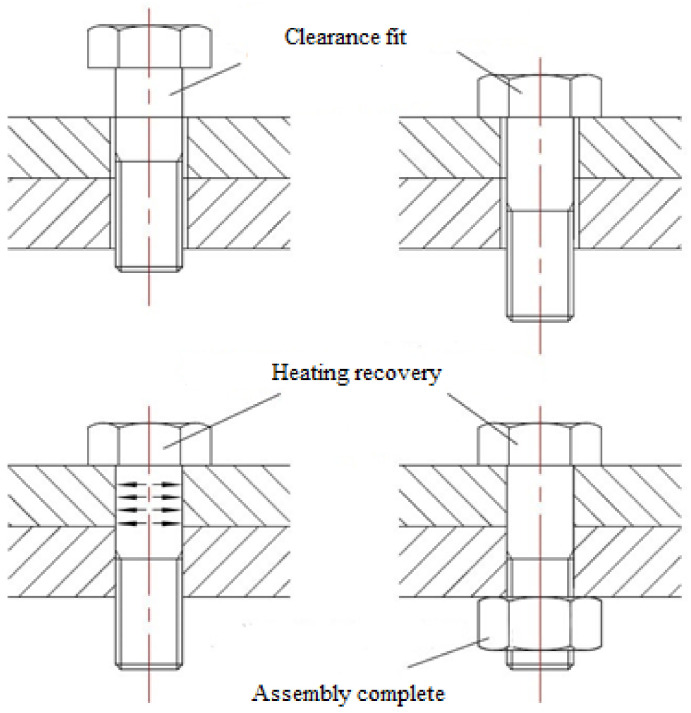
Installation principle of SMA interference connection.

The interference connection is realized by using the characteristics of SMA to recover deformation after heating. Its installation is convenient, and the defects caused by traditional interference connection, forced press-in installation, can be avoided. The principle is shown in Figure 1. When installing, the fit of the SMA bolt and connecting hole can be easily adopted with clearance fit. After installation, the connection structure is heated to make the shape of the SMA bolt recover and increase radially, so as to realize interference connection. In practical applications, the resistance heating method can be used to heat the connection structure locally. Compared with the traditional forced installation method, the SMA bolt is easy to install and has a large interference fit size, avoiding the hole wall damage and other defects caused by forced installation, and the interference effect is significantly improved.

In this paper, the design idea of interference connection based on shape recovery characteristics of SMA was proposed. The interference connection of aluminum alloy 6061-T651 with NiTiNb SMA was studied by means of a constitutive model, finite element simulation, and experimental verification.

## 2. Materials and Methods

### 2.1. Numerical Simulation of Interference Connection

#### 2.1.1. Model Creation

**Figure 2:**
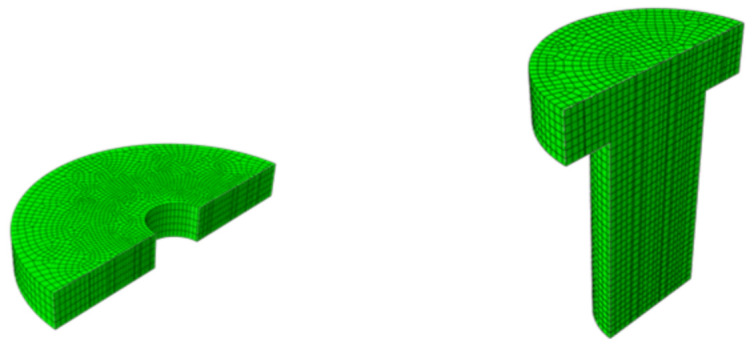
Finite element model of interference connection.

At present, there are many bolt diameters used in interference connection, such as M5, M6, and M8. The actual interference fit size of a large diameter bolt is large, so it is difficult to realize interference connection by traditional methods. It is more significant to study interference connection of the large diameter bolt. Considering the difficulty of making holes with different preinterference fit sizes in the experiment, this paper selected the M8 bolt as the research object, used aviation aluminum alloy as the connecting plate, and used ABAQUS finite element software to simulate. In order to facilitate the finite element calculation, the interference connection structure was appropriately simplified. The interaction between the bolt and the nut was not considered here, so the threaded section was ignored and regarded as the connecting shaft. The model is shown in Figure 2. Some studies have shown that the influence of interference connection is very small beyond 3 times the radius around the hole [12]. Therefore, the thickness of the aluminum alloy plate used in the model is 5 mm, and its diameter is 20 mm.

In the finite element model, the axisymmetric structure was adopted in the calculation model, and continuum axisymmetric 4 nodes reduced integration (CAX4R) was selected to divide the mesh. Considering the calculation accuracy and efficiency, the mesh refinement was carried out in the interference affected area of the connecting plate. The contact between the bolt rod and hole wall was defined as the contact pair of active and passive surfaces.

#### 2.1.2. Material Properties

The ABAQUS material library does not contain SMA materials. For the definition of SMA materials, ABAQUS provides a user-defined interface of material properties, user-defined material subroutine (UMAT). The material properties of NiTiNb SMA are defined by a constitutive model.

In this paper, the Brison model was selected, which was better used in a macroscopic phenomenological model. Its physical meaning is clear and its mathematical form is simple. It is more accurate for predicting the properties of SMA [13,14]. Assuming that the radial recovery characteristics of NiTiNb SMA are consistent with axial direction, the Brison constitutive model is expressed by

(1) $$\sigma -\sigma_{0}=E\left(\varepsilon -\varepsilon_{0}\right)+\Omega_{s}\left(\xi_{s}+\xi_{s0}\right)+\Omega_{T}\left(\xi_{T}+\xi_{T0}\right)+\Theta \left(T-T_{0}\right)$$

where E is the modulus of elasticity, Ω is the phase change tensor, Θ is the thermoelastic modulus, $\xi_{s}$ is the stress-induced martensite content, $\xi_{T}$ is the temperature-induced martensite content, and subscript 0 represents the initial state.

(2) $$E\left(\xi \right)=E_{M}\xi +E_{A}\left(1-\xi \right)$$

(3) $$\Omega \left(\xi \right)=-\varepsilon_{L}E\left(\xi \right)$$

(4) $$\Theta =\Theta_{M}\xi +\Theta \left(1-\xi \right)$$

where ξ is the percentage of martensite, the subscript *M* is martensite, and the subscript *A* is austenite.

According to the wide hysteresis characteristic of NiTiNb SMA, the martensite is induced by temperature $\Omega_{T}=0$. Since the order of magnitude of Θ is much smaller than that of E, it is treated as a constant.

The process of stress inducing martensite in NiTiNb SMA can be divided into three stages. In the initial stage of strain, the alloy undergoes elastic deformation, and the stress increases linearly with the increase in strain. With the increase in strain, the alloy enters the stage of stress-induced martensite, in which the stress almost does not change with the increase in strain. When the strain reaches the critical value, the stress begins to increase. At this time, the deformation of the alloy enters the plastic deformation stage, and the stress increases linearly with the increase in strain again.

When $T>M_{s},\varepsilon \leq 0.2\%$ or $\sigma <\sigma_{s}^{cr}+C_{M}\left(T-M_{s}\right)$,

(5) σ=Eε

When $\sigma_{s}^{cr}+C_{M}\left(T-M_{s}\right)<\sigma <\sigma_{f}^{cr}+C_{M}\left(T-M_{s}\right)$,

(6) $$\xi_{s}=\frac{1-\xi_{s0}}{2}\cos\left\{\frac{\pi }{\sigma_{s}^{cr}-\sigma_{f}^{cr}}\left[\sigma -\sigma_{f}^{cr}-C_{M}\left(T-M_{s}\right)\right]\right\}+\frac{1+\xi_{s0}}{2}$$

(7) $$\sigma -\sigma_{0}=E\left(\varepsilon -\varepsilon_{0}\right)+\Omega_{s}\left(\xi_{s}+\xi_{s0}\right)$$

where $C_{M}$ is the equivalent transformation coefficient of martensite, $\sigma^{cr}$ is the critical stress to induce martensitic transformation, and $M_{s}$ is the start temperature of martensite transformation.

Substituting $\sigma_{0}=E\varepsilon_{0},\xi_{s0}=0$ into Equation (4), we can obtain the following results:

(8) $$\sigma =E\varepsilon +\Omega_{s}\xi_{s}$$

When $\sigma \geq \sigma_{f}^{cr}+C_{M}\left(T-M_{s}\right)$,

(9) $$\sigma =\sigma_{s}+E\left(\varepsilon -\varepsilon_{L}\right)$$

When ε=16%, after unloading, the following results can be obtained:

(10) $$\varepsilon_{L}=16\%-\frac{\sigma_{16\%}}{E_{M}}=\varepsilon_{r}+\varepsilon_{rs}$$

where $\varepsilon_{r}$ is recoverable strain, and $\varepsilon_{rs}$ is the residual strain.

When ξ=1, after unloading, the following results can be obtained:

(11) $$\varepsilon_{r\max}=-\frac{\Omega }{E}$$

When ξ≤1, after unloading, the following results can be obtained:

(12) $$\varepsilon_{r}=-\xi \frac{\Omega }{E}$$

Therefore, it can be concluded that:

(13) $$\xi =\frac{\varepsilon_{r}}{\varepsilon_{r\max}}$$

The NiTiNb SMA was unloaded after tensile pre-deformation and then recovered by heating. If there is no constraint during the recovery process, the stress is 0 for the unrestrained NiTiNb SMA. From this, we can obtain the following results:

(14) $$E\left(\varepsilon_{r}-\varepsilon_{0}\right)+\Omega_{s}\left(\xi_{s}-\xi_{s0}\right)+\Theta \left(T-T_{0}\right)=0$$

Substituting $T_{0}=20$ °C, $\varepsilon_{0}=\varepsilon_{r\max}$, $\xi_{s0}=1$ into Equation (11), we can obtain the following results:

(15) $$\varepsilon_{r}=\varepsilon_{r\max}-\frac{1}{E}\left\{\Theta \left(T-A_{s}{}^{\prime }\right)+\frac{\Omega }{2}\left[\cos a_{A}\left(T-A_{s}{}^{\prime }\right)-1\right]\right\}$$

If the alloy is restrained before heating, the strain of the alloy does not change, and we can obtain the following results:

(16) $$\sigma -\sigma_{0}=\Omega_{s}\left(\xi_{s}-\xi_{s0}\right)+\Omega_{T}\left(\xi_{T}-\xi_{T0}\right)+\Theta \left(T-T_{0}\right)$$

Substituting $\xi_{s0}=1$, $\Omega_{T}=0$, $T_{0}=20$ °C into Equation (13), we can obtain the following results:

(17) $$\sigma -\sigma_{0}=\Omega_{s}\left(\xi_{s}-1\right)+\Theta \left(T-T_{0}\right)$$

When $T_{0}<T<A_{s}{}^{\prime }$,

(18) $$\sigma -\sigma_{0}=\Theta \left(T-T_{0}\right)$$

When $T>A_{s}{}^{\prime }$,

(19) $$\sigma -\sigma_{0}=\frac{\Omega_{s}}{2}\frac{\varepsilon_{r}}{\varepsilon_{r\max}}\left\{\cos\left[a_{A}\left(T-A_{s}{}^{\prime }\right)+b_{A}\sigma \right]-1\right\}+\Theta \left(T-A_{s}{}^{\prime }\right)$$

where $a_{A}$, $b_{A}$ is the material constant.

Under the action of stress, the finish temperature of austenite transformation is as follows:

(20) $$A_{f}{}^{\prime }=\frac{a_{A}-b_{A}\sigma_{AS}+b_{A}\Omega \xi_{M}+\pi }{a_{A}+b_{A}\Theta }$$

For structural parts, aluminum alloy materials can be directly defined by the material library, and the material performance parameters used in the model are shown in Table 1.

#### 2.1.3. Parameter Setting

Theoretically, the maximum recoverable strain of NiTiNb SMA is 7%. Assuming that the volume of NiTiNb SMA remains unchanged before and after pre-deformation, the maximum interference fit size is 3.2%. In order to study the interference effect of NiTiNb SMA under different interference fit sizes, an interference model between 0.5% and 3.2% was designed by changing the hole of the structural plate. The corresponding sizes and interference fit sizes are shown in Table 2.

The pretreatment condition of NiTiNb SMA is to stretch 16% at −60 °C and install at normal atmospheric temperature. Therefore, the NiTiNb SMA was stretched axially 16% at −60 °C before installation. After assembly, the temperature was raised to 150 °C, and, finally, the structure was cooled to 20 °C at normal atmospheric temperature.

### 2.2. Experiment of Interference Connection

#### 2.2.1. Materials

The NiTiNb SMA used in the experiment was prepared by a vacuum consumable melting process and then annealed at 850 °C for 24 h. The nominal composition of NiTiNb SMA was 47at% Ni, 44at% Ti, and 9at% Nb (at% represents atomic percentage). The mechanical properties of NiTiNb SMA are shown in Table 3. The aluminum alloy used in the experiment was 6061-T651 aluminum alloy.

#### 2.2.2. Tensile Pre-Deformation

The tensile pre-deformation test was carried out on an MTS-810 electro-hydraulic servo material testing machine. During the test, the axial load was controlled by displacement, and the specimen was cooled by liquid nitrogen at a loading rate of 0.5 mm/min.

**Figure 3:**
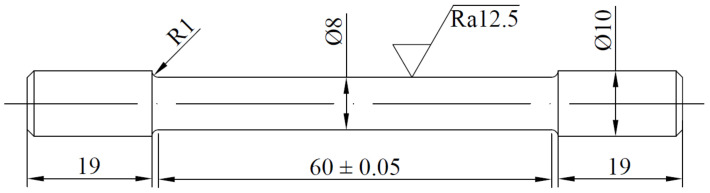
Geometric dimensions of the tensile specimen.

The material was processed into a solid tensile part for a uniaxial tensile test, and its geometric dimensions are shown in Figure 3. The total length of the uniaxial solid specimen was 100 mm, the gauge length of the specimen was 60 mm, and the diameter was 8 mm. Before deformation, the starting temperature of forward phase transformation was $M_{s}=\left(-90\pm 10\right)$ °C, the starting temperature of reverse phase transformation was $A_{s}=\left(-65\pm 10\right)$ °C, and the end temperature of reverse phase transformation was $A_{f}=\left(-25\pm 10\right)$ °C.

**Figure 4:**
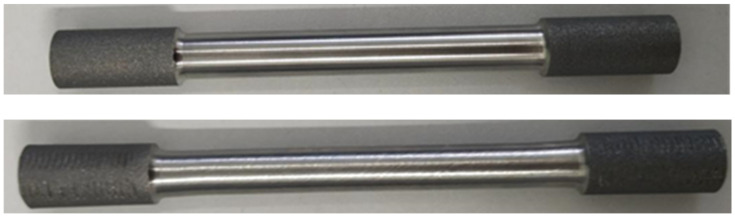
Comparison of specimens before and after tension.

The tensile specimen of NiTiNb SMA was unloaded after 16% axial tension at −60 °C, and part of the elastic recovery of the specimen strain could be achieved after the tension was completed. The tensile test piece is shown in the Figure 4. The length and diameter of the gauge section were measured after it was stable.

**Figure 5:**
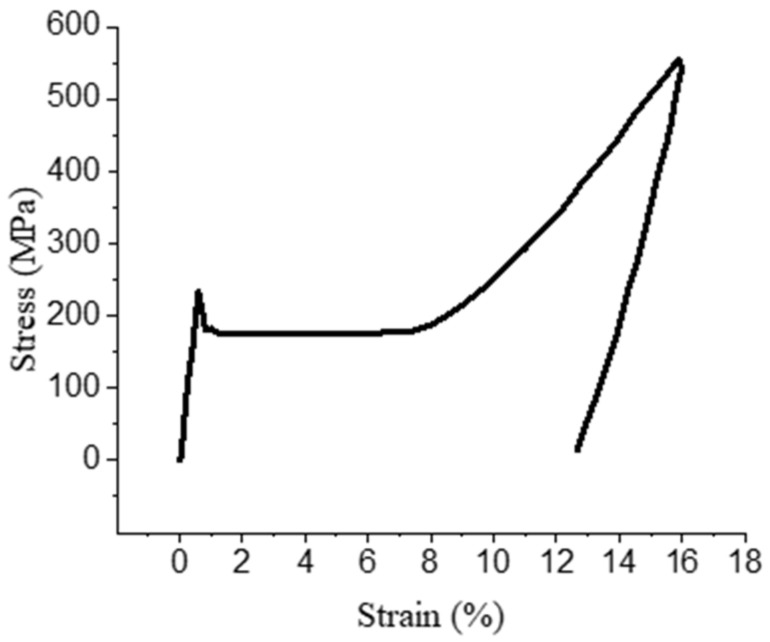
The stress-strain curve of NiTiNb SMA at −60 °C.

The stress-strain curve of NiTiNb SMA at −60 °C is shown in the Figure 5. During the tensile process, the elastic deformation first occurs, then the stress decreases and enters the phase transformation stage. The stress value in this stage is almost unchanged. With the increase in deformation, the stress begins to increase and enters the martensite elastic and plastic mixing stage. After unloading, there is elastic deformation recovery.

#### 2.2.3. Heating Recovery

**Figure 6:**
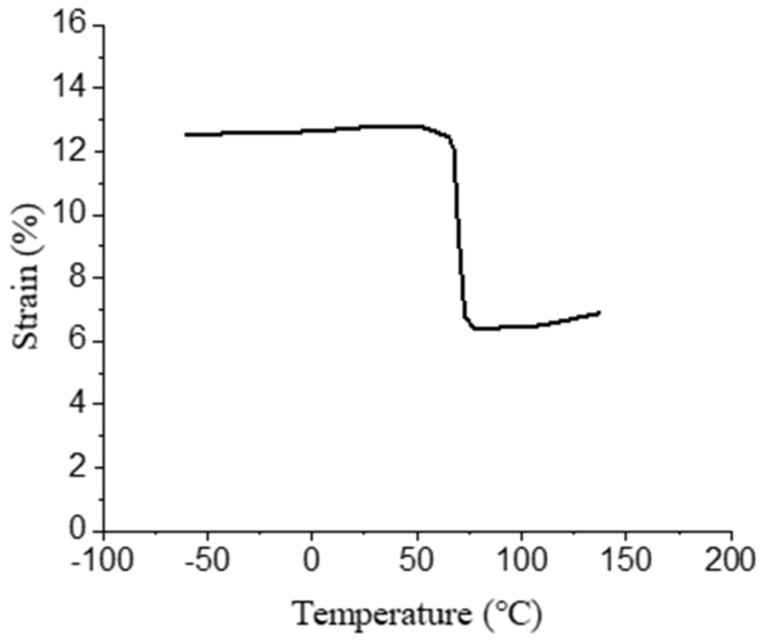
The stress-temperature curve of NiTiNb SMA heated after unloading.

After unloading, NiTiNb SMA was heated to determine its recovery temperature. The strain-temperature curve of NiTiNb SMA heated after unloading is shown in Figure 6.

After pre-deformation, the recovery temperature range of NiTiNb SMA is mainly concentrated in 60–90 °C. In order to ensure the full recovery of the specimens, they were heated to 150 °C without load using a high and low temperature test chamber. After the specimen was heated, the axial and radial recoveries were measured and the recovery rate was calculated. Six groups of tensile tests were carried out. The test results are shown in Table 4. The maximum recovery rate of the NiTiNb SMA used in this experiment is about 5.42% in the axial direction and 2.39% in the radial direction. The maximum interference fit size is 2.39%.

#### 2.2.4. Interference Connection

**Figure 7:**
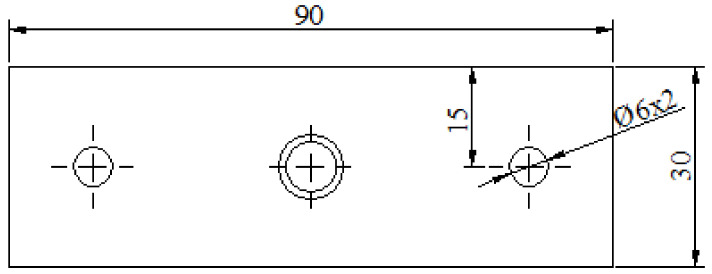
Geometric dimensions of the connection plate.

Based on the measured maximum radial recoverable strain of NiTiNb SMA specimens, the interference connection tests under different interference fit sizes were designed. The diameters of the connecting holes and the corresponding interference fit sizes are shown in Table 5. The connecting plate was made of 6061-T651 aluminum alloy with a length of 90 mm, a width of 30 mm, and a thickness of 5 mm. Its structural dimensions are shown in Figure 7.

**Figure 8:**
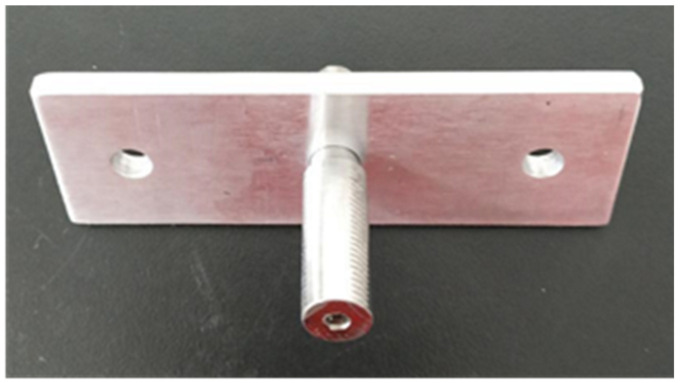
Test specimen of interference connection.

This test was only to study the interaction between the heat recovery of the bare rod and the connecting hole, and the influence of the threaded section and nut on interference connection was not considered at this time. Therefore, the threaded section was not processed. The tensile specimen could be directly cut off after pre-deformation treatment, and the interference fit size could be controlled by changing the diameter of the connecting hole. The interference connection test of each group was carried out three times. The interference connection specimen is shown in Figure 8.

#### 2.2.5. Interference Force Measurement

Because the stress and strain in the test were difficult to measure directly, the pull-out test was carried out on the test piece, and the pull-out force was used to characterize the interference force. The relationship is as follows:

(21) F=σμπDh

where D is the diameter of the hole, h is the height of the hole, and μ is the coefficient of friction.

**Figure 9:**
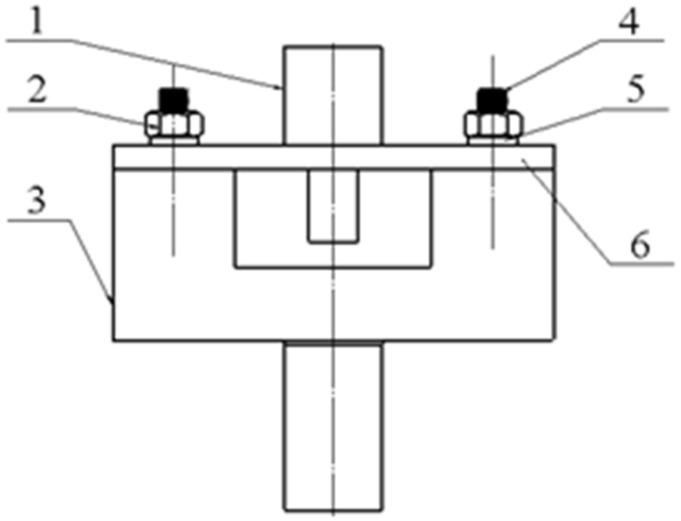
The structure of the fixture model in the pull-out test. 1. NiTiNb SMA parts; 2. nuts; 3. fixture base; 4. double-headed studs; 5. gaskets; 6. aluminum alloy structural parts.

The pull-out test is shown in Figure 9.

## 3. Results and Discussions

### 3.1. Analysis of Numerical Simulation Stress

**Figure 10:**
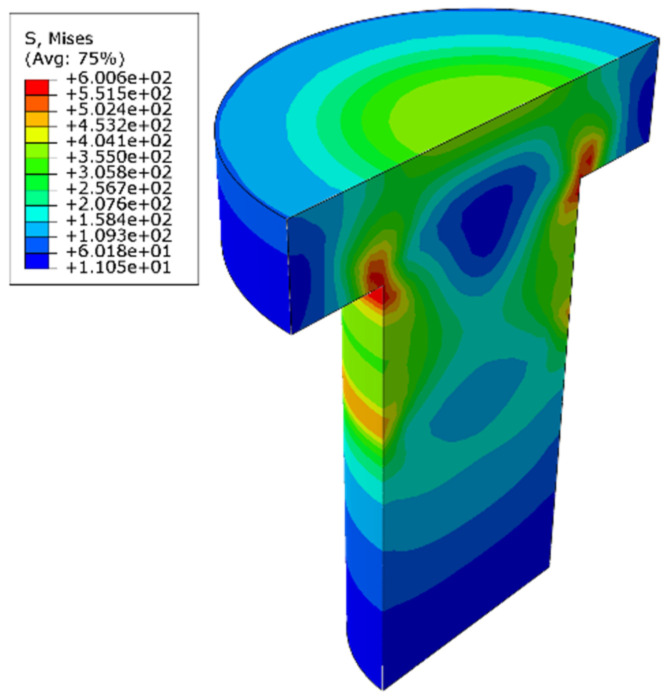
The stress distribution of the bolt with 3.2% interference.

**Figure 11:**
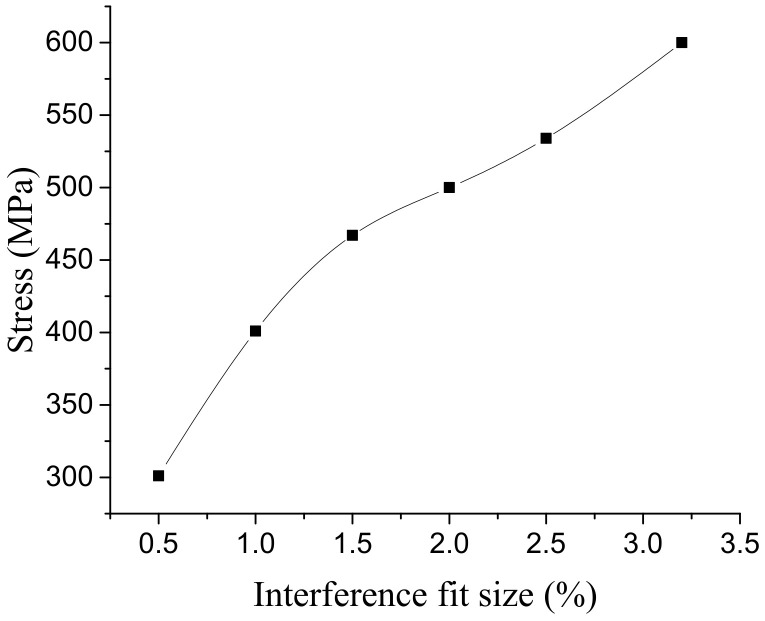
Variation of bolt maximum stress with interference fit size.

The 3.2% interference model was taken as an example to analyze. Figure 10 shows the stress distribution of the bolt with 3.2% interference. The results show that the maximum stress appeared at the root of the bolt rod, which is due to the axial restoring force of NiTiNb SMA after heating between the bolt head and the connecting plate. The material in the center of the bolt shrinks axially, and the deformation of the material at the peripheral position is blocked by the connecting plate, so there is a stress concentration here. The variation of the maximum stress of the bolt with the interference fit size is shown in Figure 11. The maximum stress value increases with interference fit size. When the interference fit size exceeds 3%, the value reaches the yield limit of the bolt rod material. In addition to the stress concentration of the bolt head, the bolt rod also forms a stress concentration at the lower outlet of the connecting plate, while the stress distribution in other parts is more uniform, and the stress value is small.

**Figure 12:**
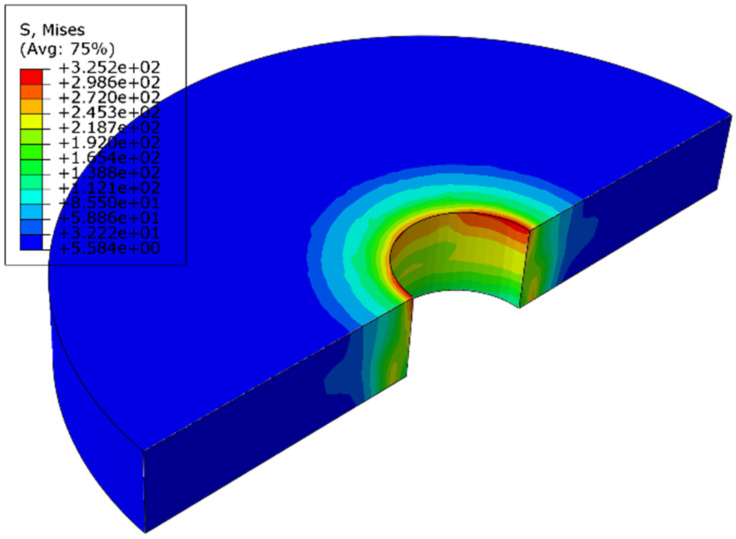
The stress distribution of the connection plate with 3.2% interference.

**Figure 13:**
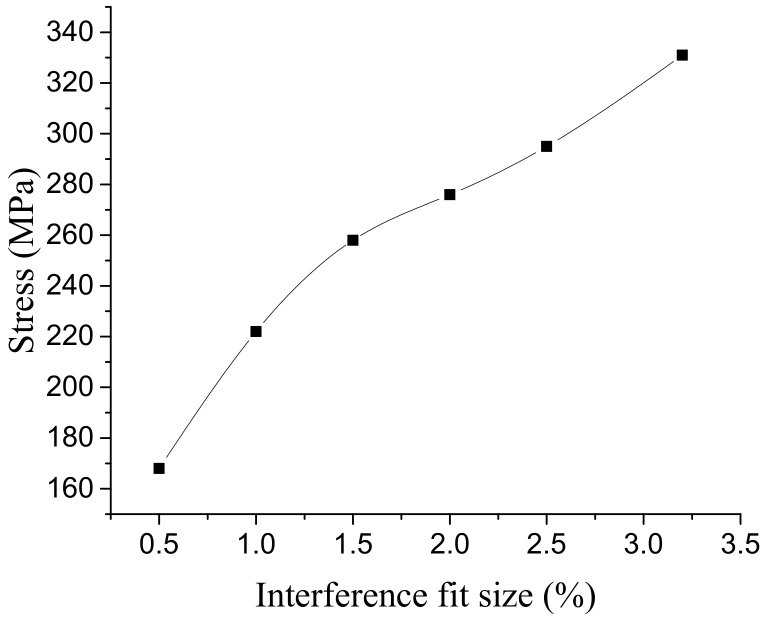
Variation of connecting plate’s maximum stress with interference fit size.

Figure 12 shows the stress distribution of the connection plate with 3.2% interference. The stress distribution on the connecting plate extended outward with the hole wall as the center. In the area near the axis of the hole, the stress value is larger and the change is obvious. In the area further from the hole axis, the stress value is gradually smaller. After exceeding a certain range, the stress value basically does not change. The larger stress occurs at the outlet and entrance of the hole, which is mainly due to the free recovery section and connection part of the bolt rod, where the amount of recovery is different. The maximum stress occurred at the hole wall near the entrance, and its value changed with the interference fit size, as shown in Figure 13. When the interference fit size is less than 1%, the maximum stress is lower than the material yield limit, and the hole wall deformation is in a state of elastic deformation; when the interference fit size is 1.5%, the maximum stress is greater than the material yield limit, and the hole wall has plastic deformation; when the interference fit size is more than 2.5%, the maximum stress value exceeds the tensile limit of the connecting plate material, which does not meet the process requirements.

**Figure 14:**
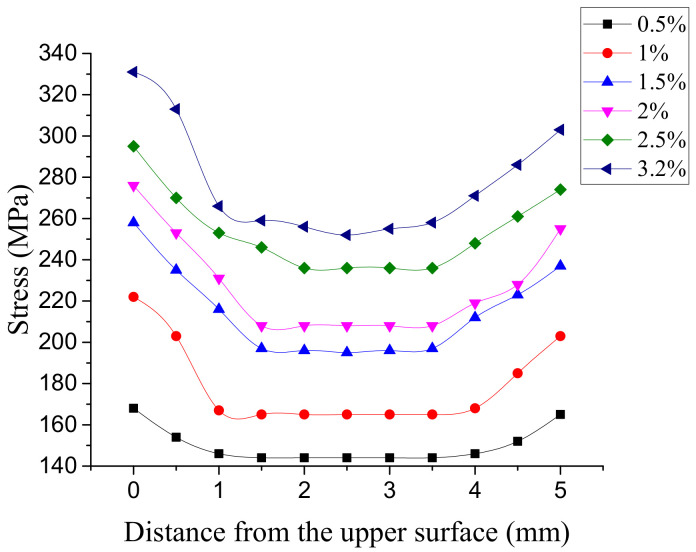
Stress distribution of the hole wall with different interference fit sizes.

The stress distribution on the hole wall under different interference fit sizes is shown in Figure 14. When the interference fit size is 0.5%, the stress distribution on the hole wall is uniform, the stress value is small, and the hole wall deformation is in a state of elastic deformation. When the interference fit size is 1%, the stress distribution on the hole wall is generally uniform, but the stress value at the hole inlet and outlet has increased to a certain extent, and the hole wall deformation is still in the elastic deformation state. When the interference fit size is 1.5%, the maximum stress value on the hole wall exceeds the yield limit of the connecting plate, the deformation of the hole wall enters the stage of elastic–plastic deformation, and the stress distribution in the remaining parts is more uniform. When the interference fit size is 2%, the stress concentration at the entrance and exit of the hole is more significant. With the increase in the interference fit size, the maximum stress value reaches the tensile limit of the connecting plate material when the interference fit size is 2.5%. When the interference fit size is more than 2.5%, the hole wall will yield as a whole and enter into a state of plastic deformation. At this time, the maximum stress value exceeds the tensile limit of the connecting plate material, which does not meet the engineering requirements. When the connection structure is under external load, the stress amplitude will increase significantly at the position of stress concentration, which can easily initiate a fatigue crack and become the source of fatigue failure. According to the above analysis, the maximum interference fit size between the NiTiNb SMA bolt and aluminum alloy is 2.5%. Using the traditional interference connection methods, the interference amount is 2% [12]. This method is superior to the traditional interference connection method.

### 3.2. Analysis of Numerical Simulation Strain

The traditional interference connection methods produce bumps in the installation process [12]. For a NiTiNb SMA interference connection, the simulation results show that there were no bumps under each interference fit size. The reason for this may be that the radial restoring force of the bolts can squeeze the hole wall of the connection plate and elastic–plastic deformation can make the hole larger, but it is different from the traditional pressure installation. The axial recovery of the bolts is relatively small, the material flow in the axial direction of the hole wall caused by friction between bolts and the hole wall is also small, and the direction of recovery is contracted inward relative to the hole mouth, so no bumps would be produced.

**Figure 15:**
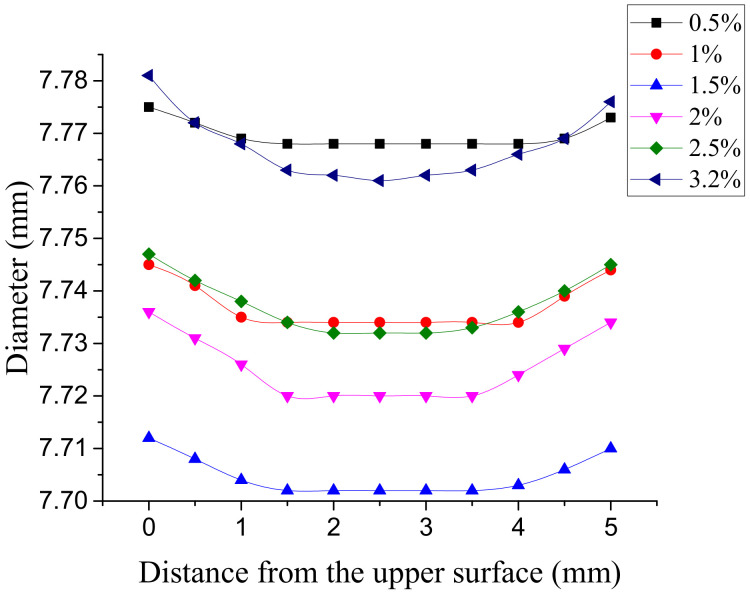
Actual aperture with different interference fit sizes.

The actual aperture of the connecting plate hole under each interference fit size is shown in Figure 15. When the interference fit size is less than 1%, the overall deformation of the hole wall is small, and the aperture difference is not obvious. When the interference fit size is more than 1%, there are two inflection points in each curve. The inflection points are close to the outlet and entrance of the hole, respectively. The greater the interference fit size, the more significant the inflection points. The distance from the upper and lower inflection points to the entrance and exit of the hole is the deformation area of the hole wall, and the part of the hole wall deformation between the two inflection points is more uniform. The larger the interference fit size, the farther the inflection point is from the orifice, and the more serious the deformation of the orifice wall.

### 3.3. Analysis of Interference Force

**Figure 16:**
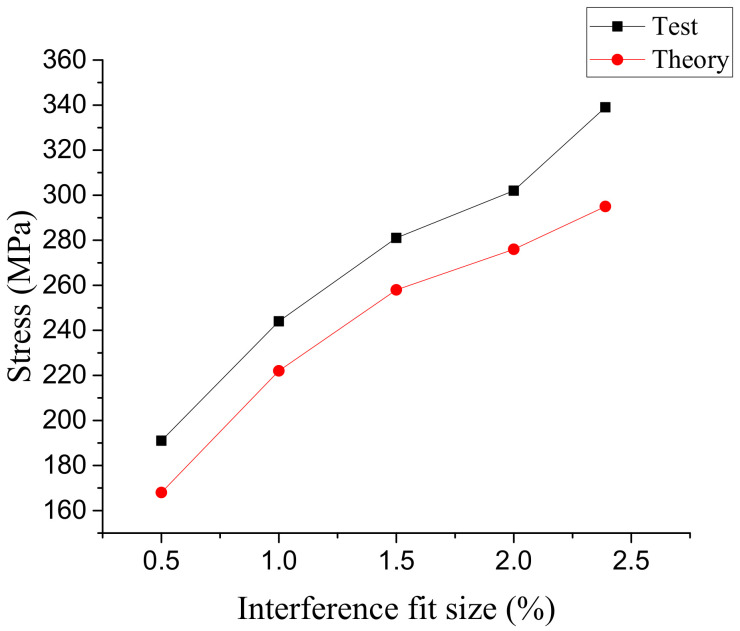
Comparison of variation of interference force with interference fit size.

The actual interference force was compared with the finite element simulation results under the same interference fit size, and the results are shown in Figure 16. It can be seen from Figure 9 that the actual interference force is higher than the result of finite element simulation under the same interference fit size, and the error is between 8% and 13%, both of which increase with the increase in interference fit size. The actual interference force is higher than the result of finite element simulation, which is mainly due to the deformation of the hole wall of the connecting hole after the interference installation, which increases the friction coefficient and leads to the increase in the pulling force. With the increase in the interference fit size, the plastic deformation of the hole wall is intensified, which causes the difference between the test results and the finite element simulation results. The reasons for the errors are as follows: (1) there are some errors between the constitutive model and the actual performance of SMA; (2) during the test, the hole wall is deformed after being squeezed, which leads to the increase in the friction coefficient, and the increase in the friction coefficient leads to the increase in pulling-out force; (3) there are some machining errors in the sample.

## 4. Conclusions

In this paper, the design idea of interference connection based on shape recovery characteristics of SMA was proposed. The interference connection of 6061-T651 aluminum alloy with NiTiNb SMA was studied by means of a constitutive model, finite element simulation, and experimental verification. The main conclusions are as follows:

(1) The results of numerical simulation show that the method of interference connection using SMA can achieve large interference fit size connection, and the maximum interference fit size with aluminum alloy connecting plate was about 2.5%. There was no bump in the resulting interference connection. There was a stress concentration at the root of the bolt, and the stress distribution of the hole wall was uniform.
(2) When the interference fit size was less than 1%, the connection hole had elastic deformation. When the interference fit size was 1.5%, the hole wall had plastic deformation. When the interference fit size was 2.5%, the maximum stress on the connecting plate was close to the tensile limit of the material. If the interference fit size continues to increase, the strength of the connection structure will be damaged.
(3) The results of the interference connection test show that there was no bump after the interference connection was completed. The interference force was calculated by pulling-off force, and the interference force increased with the increase in interference fit size. Compared with the results of finite element analysis, the change trend of the interference force is basically the same, and the error is less than 13%, which verifies the rationality of the finite element simulation.

## Figures and Tables

**Table 1:** Material parameters.

Material	Property	Value
NiTiNb	Austenite transformation A_s_′	63 °C
	Austenite transformation A_f_′	125 °C
	Maximum recoverable strain	7%
	Modulus of elasticity E	89,088 MPa
	Poisson ratio v	0.39
	Yield strength σ_s_	520 MPa
Aluminium alloy 6061-T651	Modulus of elasticity E	72,000 MPa
	Poisson ratio v	0.33
	Yield strength σ_s_	240 MPa
	Friction factor μ	0.15

**Table 2:** Size of hole and shaft with different interference fit sizes.

**Interference Fit Size**	0.5%	1%	1.5%	2%	2.5%	3.2%
Shaft d/mm	7.544					
Hole D/mm	7.752	7.714	7.676	7.638	7.601	7.544

**Table 3:** The mechanical properties of NiTiNb SMA.

Modulus of Elasticity	Yield Strength	Breaking Strength	Percentage Elongation
87 GPa	800 MPa	500 MPa	30%

**Table 4:** Size of the standard distance section of tensile specimens.

Number	Before Tension		After Tension		After Recovery		Recovery Rate	
	Length/mm	Diameter/mm	Length/mm	Diameter/mm	Length/mm	Diameter/mm	Length/mm	Diameter/mm
1	60.23	7.97	67.46	7.53	64.28	7.71	5.27	2.26
2	60.39	7.99	67.53	7.52	64.59	7.69	4.87	2.12
3	60.18	8.00	67.61	7.54	64.35	7.73	5.42	2.39
4	60.56	8.01	67.59	7.54	64.48	7.72	5.14	2.25
5	59.89	7.97	67.49	7.53	64.37	7.69	5.21	2.01
6	60.31	7.99	67.42	7.51	64.41	7.68	4.99	2.13

**Table 5:** Size of the hole and shaft with different interference fit sizes.

**Interference Fit Size**	0.5%	1%	1.5%	2%	2.39%
Shaft d/mm	7.54				
Hole D/mm	7.69	7.65	7.61	7.57	7.54

## References

[1] Taghizadeh H., Chakherlou T.N., Ghorbani H., Mohammadpour A. (2015). Prediction of fatigue life in cold expanded fastener holes subjected to bolt tightening in Al alloy 7075-T6 plate. Int. J. Mech. Sci..

[2] Croccolo D., Agostinis M.D., Ceschini L., Morri A., Marconi A. (2013). Interference fit effect on improving fatigue life of a holed single plate. Fatigue Fract. Eng. Mater. Struct..

[3] Kim S.Y., Hennigan D.J., Kim D., Seok C.S. (2012). Fatigue enhancement by interference-fit in a pin-loaded glass fibre-reinforced plastics laminate. J. Mech. Eng. Sci..

[4] Jani M.J., Leary M., Subic A., Gibson M.A. (2014). A review of shape memory alloy research, applications and opportunities. Mater. Des..

[5] Duerig T., Pelton A., Stöckel D. (1999). An overview of nitinol medical applications. Mater. Sci. Eng. A.

[6] Kajiwara S., Baruj A.L., Kikuchi T., Shinya N. (2003). Low-cost high-quality Fe-based shape memory alloys suitable for pipe joints. Proc. SPIE Int. Soc. Opt. Eng..

[7] Shishkin S.V., Shishkin S.S. (2010). The application of rivets with shape memory in aeronautical engineering. J. Mach. Manuf. Reliab..

[8] Humbeeck J.V. (1999). Non-medical applications of shape memory alloys. Mater. Sci. Eng. A.

[9] Sun L., Huang W.M., Ding Z., Zhao Y., Wang C.C., Purnawali H., Tang C. (2012). Stimulus-responsive shape memory materials: A review. Mater. Des..

[10] Otsuka K., Ren X. (2005). Physical metallurgy of Ti–Ni-based shape memory alloys. Prog. Mater. Sci..

[11] Wang M., Jiang M., Liao G., Guo S., Zhao X. (2012). Martensitic transformation involved mechanical behaviors and wide hysteresis of NiTiNb shape memory alloys. Prog. Nat. Sci. Mater. Int..

[12] Jiang J.F., Bi Y., Dong H., Ke Y., Fan X., Du K. (2014). Influence of interference fit size on hole deformation and residual stress in hi-lock bolt insertion. J. Mech. Eng. Sci..

[13] Thamburaja P. (2005). Constitutive equations for martensitic reorientation and detwinning in shape memory alloys. J. Mech. Phys. Solids.

[14] Brinson L.C., Huang M.S. (1996). Simplifications and Comparisons of Shape Memory Alloy Constitutive Models. J. Intell. Mater. Syst. Struct..

